# Clinical application of exosomes and circulating microRNAs in the diagnosis of pregnancy complications and foetal abnormalities

**DOI:** 10.1186/s12967-020-02227-w

**Published:** 2020-01-22

**Authors:** Haiou Yang, Qianqian Ma, Yu Wang, Zhenhua Tang

**Affiliations:** 1 Department of Laboratory Medicine, The International Peace Maternity and Child Health Hospital, School of Medicine, Shanghai Jiao Tong University, Shanghai, China; 2 Shanghai Key Laboratory of Embryo Original Diseases, Shanghai, China; 3 Shanghai Municipal Key Clinical Specialty, Shanghai, China

**Keywords:** Pregnant women, Exosomes, MicroRNAs, Pregnancy complications, Foetal diseases

## Abstract

During pregnancy in humans, the physiology of the mother and foetus are finely regulated by many factors. Inappropriate regulation can result in pregnancy disorders, such as complications and foetal abnormalities. The early prediction or accurate diagnosis of related diseases is a concern of researchers. Liquid biopsy can be analysed for circulating cells, cell-free nucleic acids, and exosomes. Because exosomes can be detected in the peripheral blood of women in early pregnancy, these vesicles and their contents have become the focus of early prediction or diagnostic biomarker research on pregnancy complications and foetal developmental disorders. In this review, we focus on recent studies addressing the roles of peripheral blood exosomes and circulating miRNAs in pregnancy complications and in pregnancies with abnormal foetal developmental disorders, with particular attention paid to the potential application value of exosomes and circulating miRNAs as disease-specific biomarkers.

## Background

Pregnancy is the process by which women give birth to progeny. Most pregnant women have a normal pregnancy and delivery; however, some experience abnormalities during pregnancy that affect their health or the health of the foetus, such as complications and foetal development and growth abnormalities [1]. Pregnancy complications refer to the condition of the woman due to pregnancy, including gestational diabetes, gestational hypertension and pre-eclampsia, preterm birth, early abortion, and foetal growth restriction, among others. Abnormal foetal development and growth diseases include neural tube defects, congenital heart disease, and multiple malformations [1]. Previous studies have revealed that pregnancy complications or adverse outcomes during pregnancy can increase the risk of certain diseases, such as diabetes, after maternal delivery and affect the growth and development of newborns [2, 3]. At present, the early diagnosis of pregnancy complications and abnormal foetal development diseases relies on routine haematology screening and ultrasound examination. For example, blood glucose monitoring during pregnancy can be used for diagnosing gestational diabetes; blood pressure measurement and urine protein detection can be used for screening pregnancy hypertension and pre-eclampsia, among others, and colour Doppler ultrasound is often used for monitoring foetal growth and development. However, when complications and structural abnormalities in pregnant women or foetuses are detected, the optimal clinical intervention time has generally been missed or irreversible damage to the foetus has occurred. Amniotic fluid detection is a highly specific diagnostic method for screening foetal developmental abnormalities. Nonetheless, amniotic fluid detection is an invasive diagnosis and requires certain indications, which greatly limit its clinical application in the field of early diagnosis. Currently, samples that are easily accessible in laboratories mainly include blood and urine. As blood is widespread throughout the body, identification of novel biomarkers from blood that can be used for the early diagnosis of pregnancy complications or foetal abnormalities is urgently needed.

The maternal peripheral blood contains a variety of factors that reflect pregnancy status, such as blood glucose, glycocholic acid, soluble Fms-like tyrosine kinase-1, and circulating foetal DNA [4, 5]. Recent studies have confirmed that exosomes and circulating miRNAs exist in peripheral blood and are involved in a variety of physiological and pathological processes [6]. Moreover, studies have revealed that exosomes can be detected as early as in the late first trimester of normal pregnancy and can increase throughout normal pregnancy [7–9]. Exosomes have a double-layered lipid membrane, which is not easily degraded by enzymes in bodily fluids, and exosomes originate from different host cells and can specifically reflect the biological information of the host cells. Furthermore, exosomes contain numerous biologically active molecules, such as nucleic acids and proteins derived from host cells, which can play different biological functions. Therefore, exosomes, especially their contents, have great appeal for in vitro diagnostics. In this review, we focus on the role of circulating miRNAs and exosomes in pregnancy complications and foetal diseases.

## Exosomes and circulating miRNAs

Exosomes are nanosized extracellular vesicles (30–100 nm) that are released from many cell types, including mast cells, endothelial cells, fibroblasts, mesenchymal stem cells and tumour cells [10, 11]. These vesicles are widely present in various natural body fluids, such as peripheral blood, urine, amniotic fluid, milk and bronchoalveolar lavage fluid. Exosome formation is different from endocytosis and exocytosis and is characterized by endosomal origin with formation through the inward budding of multivesicular bodies and release into the extracellular space via exocytosis [12]. Exosomes selectively package a large number of biologically active molecules, such as nucleotides (including nucleotides, miRNAs, long-non-coding RNA and DNA), proteins and lipids, from donor cells [6, 11–15]. The plasma membrane of exosomes is rich in heat shock proteins, TSG101, Alix, Foltillin, Rab and four transmembrane superfamily members: CD63, CD9, and CD81 [12, 13]. The specific membrane markers of donor cells can also be detected in exosome membranes. For example, lymphocyte-derived exosomes contain CD3, platelet-derived exosomes contain CD41, monocyte-derived exosomes contain CD14, and erythrocyte-derived exosomes contain CD235a/b [10, 16, 17]. Exosomes exert their biological functions mainly through three mechanisms: (1) exosomes enter recipient cells, release their contents, and re-form multivesicular bodies; (2) the exosome plasma membrane fuses with the receptor cell plasma membrane, and its contents are released into the recipient cell; and (3) the ligands on exosomes bind to specific receptors on the receptor cell membrane, initiating signal transduction. Exosomes can also be transported into recipient cells via endocytosis. Therefore, exosomes have powerful biological functions, mediate cell–cell communication through molecular transport similar to a “boat”, and participate in many biological and pathological processes, including cardiovascular development, infectious diseases, autoimmune diseases, oncogenesis and metastasis [6, 11, 18–24].

The peripheral blood-derived exosomes of pregnant women can be classified into different types according to their diverse origins. Most of these vesicles are derived from maternal cells, such as B cells, T cells, neutrophils, and endothelial cells [16, 20]. A small number of these vesicles originate from cells that comprise the placenta, including syncytiotrophoblasts, cytotrophoblasts, extravillous trophoblast cells, and placental vascular endothelial cells, with syncytiotrophoblasts being the main producers of placental exosomes [15, 22, 25, 26]. Studies have shown that placental alkaline phosphatase can be used as a specific antigen and can usually be detected to evaluate the concentration of placental exosomes in maternal serum [7, 27]. Most previous studies have focused on the analysis of total exosomes or placental exosomes in the peripheral blood of pregnant women, and differences in the diverse origins of exosomes have been poorly studied.

In addition, a variety of circulating miRNAs are included in the peripheral blood of pregnant women. miRNAs, small non-coding RNAs, are relatively conserved in evolution [28]. Most miRNAs are transcribed as precursors (either pre-miRNA or pre-mRNA) before capping and polyadenylation. In the nucleus, the primary transcripts are processed by a protein complex that contains the RNase IIIenzyme Drosha12 and DGCR8 as core components. The last step of pre-miRNA processing involves Drosha/DGCR8 complex-mediated formation of hairpin-shaped pre-miRNAs. These pre-miRNAs are exported from the nucleus to the cytoplasm, followed by further processing by RNase III Dicer, resulting in the formation of double-stranded miRNA. One strand becomes the mature miRNA, whereas the other strand is most often rapidly degraded. The mature single-stranded miRNA together with the Argonaute protein form an RNA-induced silencing complex that interacts with mRNAs. Target mRNAs are recognized through the miRNA “seed sequence”, to induce transcriptional instability or inhibit translation. miRNAs can be secreted by different types of cells, including tumour cells and placental cells. miRNAs exist in free form or are encapsulated into extracellular vesicles, such as exosomes, and these molecules participate in normal physiological regulation as well as pathogenesis of a variety of diseases, including organ development regulation, immune regulation, oncogenesis and metastasis [20, 22, 26, 29–31].

## Exosomes and circulating miRNAs in pregnancy complications

During normal pregnancy, the concentration of peripheral blood exosomes increases in a time-dependent manner until term, and this increase is significantly greater in pregnancy complications than in normal pregnancy [7, 32, 33]. Gestational age and pregnancy status have been identified as significant factors contributing to variations in peripheral blood exosomes. After delivery, the concentration of blood exosomes returns to nonpregnancy levels within 48 h [34]. The peripheral blood exosomes in pregnant women contain a complex cargo of RNA, proteins and lipids that mediate different signal transduction pathways between cells and exert diverse biological functions, such as inhibiting immune cell activity, promoting maternal foetal immune tolerance and vascular smooth muscle cell migration, recasting uterine spiral arteries and enhancing placental antiviral defences through autophagy [6, 31, 35–38]. As maternal-derived exosomes in peripheral blood translocate to the foetus through the placental villous space and exosomes of placental origin can be detected in maternal circulation from the 6th week of gestation, measurement of peripheral blood exosomes in pregnant women may contribute to the prediction or diagnosis of pregnancy-related diseases, such as placental dysfunction [7, 22, 23, 33, 39–41].

### Peripheral blood exosomes and circulating miRNAs in gestational hypertension

Pregnancy-induced hypertension refers to one of four conditions: pre-existing hypertension, gestational hypertension and pre-eclampsia, pre-existing hypertension plus superimposed gestational hypertension with protein-urea and unclassifiable hypertension. One of the most common diseases is pre-eclampsia, which affects 3%–5% of pregnancies and is one of the main causes of maternal and neonatal mortality and morbidity [42]. When left untreated, pregnant women with pre-eclampsia may have severe complications related to foetal growth restriction and preterm birth [43]. Children born to pregnant women with pre-eclampsia have an increased risk of cerebral palsy and bronchopulmonary dysplasia [43]. At present, the exact pathogenesis of pre-eclampsia is not fully understood.

Based on multiple studies, the concentrations of total peripheral blood exosomes, especially placental-derived exosomes, in pregnant women with hypertensive disorders are significantly elevated and correlate positively with disease severity compared with normal pregnancy, and are thus considered potential biomarkers for pre-eclampsia [9, 25, 44, 45].

As exosomes play roles in intercellular information transmission, the bioactive molecules that exosomes selectively package from the original cells become key factors in the biological functions of these vesicles through altered gene expression. The contents of peripheral blood exosomes, especially miRNAs, exhibit differential expression profiles between normal pregnancies and preeclamptic pregnancies and in different types of hypertension [46]. In addition, placental release of miRNAs into the maternal circulation mainly occurs via the exosomal pathway, but these molecules can also be secreted in apoptotic bodies and as protein-bound miRNAs (including circulating miRNAs). Studies have confirmed that more miRNAs, including miR-210, miR-152, and miR-411, among others, are differentially expressed in preeclamptic placentas compared with normal placentas, and several miRNAs are clustered on human chromosomes 13, 14 and 19 [46]. Moreover, plasma exosomal miR-210 and chromosome 19 miRNA cluster (C19MC) microRNAs, including miR-517-5p, miR-520a-5p, and miR-525-5p, are differentially expressed in pre-eclampsia compared to normal pregnancy [46–50]. Further studies have revealed that placental-derived exosomes mediate placental formation and various physiological and pathological placenta-maternal processes, such as immune regulation, antiviral activity, and anti-angiogenesis, and participate in the occurrence of some pregnancy diseases, including placental dysfunction, pregnancy-induced hypertension and pre-eclampsia [31, 38, 41]. Therefore, the detection of peripheral blood exosomes and circulating miRNAs in pregnant women is expected to identify early diagnostic markers for different types of pregnancy-induced hypertension disorders.

### Peripheral blood exosomes and circulating miRNAs in gestational diabetes

Gestational diabetes mellitus (GDM), one of the most common complications of pregnancy, is defined as glucose intolerance with onset or first recognition during pregnancy. GDM can increase maternal and foetal susceptibility to certain diseases, including perinatal complications, postpartum cardiovascular disease, macrosomia, premature births, malformations and diabetes.

The concentrations of exosomes and placenta-derived exosomes in the peripheral blood of GDM pregnancies are significantly higher than those in normal pregnancies [8]. Gestational age and pregnancy status have been identified as significant factors contributing to variations in plasma exosome concentration. Although exosome concentrations increase during gestation in both normal and GDM pregnancies, the extent of this increase is significantly greater in GDM. Indeed, a mouse model confirmed that GDM pregnancies are associated with a several-fold increase in the concentration of exosomes in maternal plasma compared with normal pregnancy, which is consistent with the results of human studies.

The discovery of pregnancy-related miRNAs and their respective characterization will provide us with important information regarding their function in maternal and placental metabolic regulation [51]. Analysis of the circulating miRNA profiles in peripheral blood has revealed that miR-1275, miR-16-5p, miR-17-5p, miR-19a-3p, miR-19b-3p, miR-20a-5p, miR-195-5p and miR-3664-3p are significantly upregulated in GDM pregnancies compared with those in normal pregnancies. In contrast, miR-29a, miR-132, miR-142-5p, miR-222 and miR-4666a-3p were found to be downregulated in women affected by GDM [51–57]. RNA sequencing has revealed that miR-138-5p is significantly downregulated in GDM placentas [53]. A recent study reported that ten exosomal miRNAs in serum were present at significantly higher levels in GDM cases than in controls [56]. Bioinformatics analysis and cell biology experiments have further confirmed that differential miRNAs are associated with fatty acid biosynthesis and metabolism, pro-inflammatory immune responses, trophoblast proliferation and differentiation, insulin secretion and glucose transport [35, 51, 58]. Thus, circulating miRNAs in plasma and exosomal-derived miRNAs in the peripheral blood of pregnant women play a certain role in physiological regulation in normal pregnancy and may participate in the pathogenesis of gestational diabetes.

### Circulating and exosomal-derived miRNAs in premature delivery

Preterm delivery is considered to be one that occurs before 37 complete weeks of gestation. Polycyesis and infection during pregnancy are thought to be the main causes of preterm birth. Smoking, drinking, mental stress and premature birth history can increase the risk of preterm birth. Premature babies have high rates of various diseases, including respiratory illnesses, retinopathy of prematurity, intracranial haematoma, cerebral palsy and even death. Thus, identifying and monitoring molecular biomarkers to predict the risk may help clinicians to reduce or prevent preterm birth.

Proteins have long been studied as biomarkers of preterm delivery, and using mass spectrometry, many factors from the peripheral blood or amniotic fluid of early pregnant women have exhibited a unique ability to predict risk for preterm birth and premature rupture of membranes [36, 59, 60]. In recent years, circulating miRNAs and exosomes have attracted much attention for their potential as disease biomarkers. The use of miRNA array analysis has revealed that circulating miRNAs, including miR-302b, miR-548, and miR-1253, are downregulated and that miR-223 is upregulated in plasma from those with spontaneous preterm birth compared to those with normal pregnancy [61]. Moreover, high-throughput sequencing platform results suggest that the miRNA content of circulating exosomes is significantly different in pregnant women with preterm birth or spontaneous preterm labour compared to those with normal pregnancy [40, 61, 62]. Thus, maternal plasma circulating miRNAs or extracellular vesicle miRNAs can be used to reflect placental pathological changes and as biomarkers to predict preterm birth.

### Peripheral blood exosomes and circulating miRNAs in foetal growth restriction

Foetal growth restriction (FGR) is characterized as the failure of a foetus to reach its growth potential and is usually referred to as a pathological condition. FGR is one of the main causes of perinatal morbidity and mortality and is typically caused by foetal factors, maternal factors and placental dysfunction. Due to the lack of effective therapy, early diagnosis for effective prevention and induced delivery is essential to reduce FGR.

Using quantum dots coupled with CD63 and placental-type alkaline phosphatase antibodies, the concentrations of total plasma exosomes and placental-derived exosomes in pregnant women with FGR are similar to those in normal pregnancy [63]. However, the ratio of placental-derived to total exosomes was significantly reduced in the FGR group. These results suggest that placental-derived exosomes may reflect foetal growth status and serve as a potential biomarker for FGR.

In addition to placental-derived exosomes, placental-related miRNAs exist in the circulation of pregnant women. The most frequently studied miRNA is the placental-specific C19MC, which is expressed only in placental and undifferentiated cells [37, 48–50]. Approximately 30% to 40% of miRNAs carried by placental-derived exosomes and in placental tissue are located in the C19MC. Placental-derived miRNAs are released into the peripheral blood of pregnant women, gradually increase with gestation and are rapidly cleared from the circulation after delivery. A variety of circulating miRNAs, including miR-103a-3p, miR-126-3p, miR-195-5p and miR-499a-5p, also show a trend toward downregulation in pregnant women with FGR. Moreover, many miRNAs, e.g., miR-518b, miR-1323, miR-520h and miR-519d, are differentially expressed in placental tissues from women affected FGR [64–66]. Thus, peripheral blood exosome-derived miRNAs in pregnant women may reflect placental function, and placenta-specific miRNAs can be used as biomarkers for pregnancy-related diseases.

## Research progress on peripheral blood exosomes and circulating miRNAs in pregnant women in foetal diseases

The concentration of exosomes in the circulation of pregnant women gradually increases with the progression of gestation. Studies have confirmed that maternal exosomes located in the placenta are able to cross the placental barrier and infiltrate embryonic organs or tissues [67]. Based on multicolour flow cytometry, the vast majority of total exosomes are maternal-derived (released from intravascular cell types); the second most abundant group are unidentified orphan exosomes, and placental-derived exosomes comprise the smallest group of circulating exosomes [10, 17]. Furthermore, the DNA of placental origin packaged in exosomes can be isolated from the plasma and serum of pregnant women, and cell-free nucleic acids in the circulation of pregnant women can be used in prenatal diagnosis and pregnancy-associated diseases [68]. Thus, detection of the concentration of circulating exosomes and circulating nucleic acids in pregnant women can help in monitoring gestation and placenta-related diseases and can be used to help assess the growth and development of the foetus for noninvasive prenatal diagnosis.

### Circulating miRNAs in pregnant women with Down’s syndrome

Down’s syndrome, also called trisomy 21, primarily manifests as mental retardation, and there is currently a lack of effective treatment. International screening methods include traditional Down’s screening and noninvasive prenatal genetic testing; the former involves early pregnancy Down’s screening combined with neck transparent layer thickness and mid-term Down’s screening.

An analysis of 14 miRNAs encoded by chromosome 21 showed that miR-125b-2, miR-155 and miR-3156 isolated from amniotic fluid are present in significantly higher levels in pregnant women with Down’s syndrome foetuses [69]. Additionally, microarray-based genome-wide expression profiling has revealed that miR-1973 and miR-3196 are expressed at higher levels in trisomy 21 placentas than in euploid placentas [70]. Moreover, a comprehensive analysis of circulating miRNA expression levels to identify a prenatal peripheral blood miRNA signature in pregnant women with Down’s syndrome foetus demonstrated that miR-15a, let-7d, miR-23a, miR-99a, miR-142, miR-191, miR-199 and miR-3156 are expressed at higher levels than in pregnant women with normal foetuses [70–72]. These findings validate that the study of circulating miRNAs in the peripheral blood of pregnant women can help to identify screening biomarkers for Down’s syndrome.

### Circulating and exosomal-derived miRNAs in pregnant women with congenital heart disease

Congenital heart disease (CHD) includes a large number of cardiac malformations and dysfunctional diseases caused by cardiac and vascular structural malformations or dysfunctions. Through whole-genome and -exome sequencing of CHD families or sporadic cases, many genes have been revealed to participate in foetal cardiac development regulatory networks. Nonetheless, the pathogenesis of most cases remains unclear, and there may be other regulatory mechanisms, such as those involving miRNAs, contributing to the pathogenesis of CHD.

Maternal diabetes mellitus can increase the risk of foetal CHD. Injection of exosomes isolated from the circulation of diabetic mice into normal pregnant mice reportedly increased the risk of CHD in their offspring [67]. Further studies revealed that diabetic pregnant mice display significant changes in exosomal miRNA profiles compared to normal pregnant mice [73, 74]. Additionally, significant differential expression of more than 100 circulating miRNAs, including miR-34a, miR-142-5p, miR-1275, miR-4666a-3p and miR-3664-3p, in the peripheral blood of pregnant women carrying a foetus with abnormal heart development compared to women with normal pregnancy has also been reported [57, 75, 76]. These differentially expressed miRNAs are functionally predicted to be involved in the regulation of foetal heart development by bioinformatics analysis [24, 75–77]. Therefore, these results confirm that circulatory exosomes or circulating miRNAs in pregnancy participate in the regulation of foetal heart development, providing new insight into CHD diagnosis, prevention and even treatment.

### Circulating miRNAs in pregnant women with neural tube defects

Neural tube defects (NTDs) are a group of congenital birth defects in the central neural system, mainly caused by failures in neural tube closure during neurulation in early embryonic development, including anencephaly, spina bifida, and encephalocele, among others. Although NTD pathogenesis is still not fully understood, it is thought to be multifactorial in origin, involving genetic and environmental factors. Epigenetic modifications, such as DNA methylation and histone modifications, folate metabolites and related enzymes, are involved in the pathogenesis of NTDs.

Studies on the correlation between circulating exosomes and pregnant women with NTD foetuses have not yet been reported. Several studies have revealed that the methylation status of some distinct miRNAs is closely related to the occurrence of NTDs [78]. Yuan and colleagues revealed six miRNAs (miR-142-3p, miR-144, miR-720, miR-575, miR-765, miR-1182 and miR-1275) showing significant changes in expression in the serum of pregnant women with NTD foetuses, and combining these miRNAs generated a valuable result in an area under the receiver operating characteristic curve analysis [79]. These findings highlight the clinical potential of circulating miRNAs as noninvasive biomarkers for diagnosing NTDs.

## Conclusions

Pregnancy complications and birth defects seriously affect the health of women and foetuses. Currently, diagnoses for these disorders rely on conventional screening or diagnostic methods, and there is a lack of biomarkers with early predictive value. With the development of noninvasive diagnoses, exosomes and circulating miRNAs in the peripheral blood of pregnancies have garnered much attention for their potential as diagnostic biomarkers for pregnancy-related diseases.

Exosomes serve as carriers of cell-to-cell information. Through their contents, such as DNA, RNA, proteins and lipids, exosomes are involved in mediating a variety of biological functions, such as apoptosis, cell activation, proliferation, differentiation, adhesion, and migration, and thus participate in the occurrence and metastasis of tumours, autoimmune diseases, nervous system diseases and cardiovascular diseases. Exosomes can be detected in the peripheral circulation of pregnant women during the first trimester (> 6 weeks) [33]. Based on their wide distribution, easy acquisition and good stability, much attention has been given to the role of these vesicles as diagnostic biomarkers for pregnancy complications and foetal dysplasia. The exosome concentration in the peripheral blood of pregnant women is closely related to the pregnancy process and pregnancy complications, but there are few studies on abnormal foetal development. In general, abnormalities in peripheral blood exosome concentrations in pregnant women can to some extent reflect the risk of pregnancy complications, though there is no study comparing differences in the concentrations of peripheral blood exosomes between different types of pregnancy complications.

A large number of studies focusing on the contents of exosomes and circulating miRNAs have revealed that many miRNAs are associated with pregnancy complications and abnormal foetal development. However, the miRNAs identified from existing studies usually lack consistency and reproducibility, which may be caused by the following: (1) different periods of gestation, as the miRNA profiles of exosomes in the peripheral blood of pregnant women may differ in the first, second and third trimesters; (2) different analysis methods, such as miRNA-array or RNA-sequencing, which have been used to analyse circulating miRNAs or exosome-derived miRNAs, though the results obtained were not well unified; (3) different types of samples used (serum vs. plasma vs. placenta); and (4) different methods for separating and purifying exosomes. Knowledge regarding the biological characteristics of and separation techniques for exosomes will increase as methods mature. Detection techniques based on peripheral blood exosomes mainly include transmission electron microscopy, flow cytometry, nanoparticle tracer technology, adjustable resistance pulse analysis and enzyme-linked immunosorbent assay. Different detection methods result in different separation purities [10, 27, 80–83]. In addition, existing studies consider the total concentration of peripheral blood exosomes in pregnant women, and the origin of exosomes from different types of cells needs to be further illustrated. Therefore, the abovementioned factors cause great bias in the study of circulating miRNA profiles and exosomal miRNAs of pregnancy complications and foetal developmental disorders, greatly hindering the clinical application of circulating exosomes or circulating miRNAs in assessing pregnancy complications and foetal development abnormalities. Regardless, results to date provide insight into identifying candidate biomarkers for pregnancy complications and foetal developmental disorders.

With the rapid development of medical science and technology, collecting whole gestation data during pregnancy and different types of samples will help in the development of predictive models of pregnancy complications and abnormal foetal developmental disorders.

## Data Availability

Not applicable.

## Abbreviations

miRNA: microRNA
DGCR8: DiGeorge syndrome critical region gene 8 protein
GDM: gestational diabetes mellitus
FGR: foetal growth restriction
C19MC: chromosome 19 miRNA cluster
CHD: congenital heart disease
NTDs: neural tube defects
